# Radioprotective potential of probiotics against gastrointestinal and neuronal toxicity: a preclinical study

**DOI:** 10.1007/s12094-023-03184-8

**Published:** 2023-04-18

**Authors:** Babu Santhi Venkidesh, Saligrama R Shankar, Rekha Koravadi Narasimhamurthy, Satish Bola Sadashiva Rao, Kamalesh Dattaram Mumbrekar

**Affiliations:** 1 Department of Radiation Biology & Toxicology, Manipal School of Life Sciences, Manipal Academy of Higher Education, Manipal, 576104 India; 2 Manipal School of Life Sciences, Manipal Academy of Higher Education, Manipal, 576104 India; 3 Manipal Academy of Higher Education, Manipal, 576104 India

**Keywords:** Radiation, Gastrointestinal toxicity, Neuroinflammation, Probiotics, Radioprotection, Neurotoxicity

## Abstract

**Purpose:**

Radiotherapy is a critical component of cancer treatment, along with surgery and chemotherapy. Approximately, 90% of cancer patients undergoing pelvic radiotherapy show gastrointestinal (GI) toxicity, including bloody diarrhea, and gastritis, most of which are associated with gut dysbiosis. In addition to the direct effect of radiation on the brain, pelvic irradiation can alter the gut microbiome, leading to inflammation and breakdown of the gut–blood barrier. This allows toxins and bacteria to enter the bloodstream and reach the brain. Probiotics have been proven to prevent GI toxicity by producing short-chain fatty acids and exopolysaccharides beneficial for protecting mucosal integrity and oxidative stress reduction in the intestine and also shown to be beneficial in brain health. Microbiota plays a significant role in maintaining gut and brain health, so it is important to study whether bacterial supplementation will help in maintaining the gut and brain structure after radiation exposure.

**Methods:**

In the present study, male C57BL/6 mice were divided into control, radiation, probiotics, and probiotics + radiation groups. On the 7^th^ day, animals in the radiation and probiotics + radiation groups received a single dose of 4 Gy to  whole-body. Posttreatment, mice were sacrificed, and the intestine and brain tissues were excised for histological analysis to assess GI and neuronal damage.

**Results:**

Radiation-induced damage to the villi height and mucosal thickness was mitigated by the probiotic treatment significantly (*p* < 0.01). Further, radiation-induced pyknotic cell numbers in the DG, CA2, and CA3 areas were substantially reduced with bacterial supplementation (*p* < 0.001). Similarly, probiotics reduced neuronal inflammation induced by radiation in the cortex, CA2, and DG region (*p* < 0.01). Altogether, the probiotics treatment helps mitigate radiation-induced intestinal and neuronal damage.

**Conclusion:**

In conclusion, the probiotic formulation could attenuate the number of pyknotic cells in the hippocampal brain region and decrease neuroinflammation by reducing the number of microglial cells.

**Supplementary Information:**

The online version contains supplementary material available at 10.1007/s12094-023-03184-8.

## Introduction

Over the past few decades, radiotherapy has been an essential component of the therapeutic arsenal for both the curative and palliative management of cancer patients. More than 50% of newly diagnosed cancer patients receive radiotherapy, and 60% with curative intent [1]. Radiation exposure to the brain has a diverse impact on behavior and cognitive functions in a time and dose-dependent manner [2]. Pelvic radiotherapy is applied to treat cancers in the pelvic region, including genitourinary, gynecologic, anorectal origin, and localized prostate cancer. As a result, the radiation field for abdominopelvic malignancies invariably comprises relatively considerable portions of the healthy intestine. The ionizing radiation damages the mucosal surface of the gastrointestinal tract, resulting in radiation-induced bowel injury [3, 4]. High doses of ionizing radiation can have immediate and long-term effects on GI, such as villous destruction, inflammation, discomfort, ulcers, and bleeding [5]. Besides, the intestinal epithelial barrier may be damaged as a result of the cytotoxic effects of radiation exposure, which makes the barrier more permeable to luminal microorganisms and causes immunological reactions [6, 7]. Recently, the impact of the microbiota on the host’s recovery following whole-body irradiation is gaining more attention [8]. Individual differences exist in the composition of the gut microbiome and these are affected by various genetic and environmental factors, including nutrition, medicines, age, and individual health. Intestinal dysbiosis is linked to various illnesses, such as inflammatory bowel diseases and metabolic diseases, including obesity and type II diabetes, and neurological disorders [9]. For example, ammonium produced by urease-producing bacteria increases blood ammonia levels causing astrocytic injury and cerebral edema leading to CNS damage [10]. Probiotics are live microorganisms meant to improve one’s health, whether taken orally or topically [11] and are one of the strategic approaches to minimize radiation-induced GI toxicity [12, 13]. Probiotics have been shown to improve cognitive impairment and depressive disorder in humans and experimental animal models through the gut-brain axis [14]. Further, probiotics are also explored in treating neurodegenerative diseases such as Alzheimer’s [15] and Parkinson’s [16]. The radiation-induced intestinal damage decreases gut integrity and facilitates the bacterial content to reach the brain more effectively. Further, gut dysbiosis can create abnormal gut–brain axis communication and causes an increase in inflammation and oxidative damage, as well as an imbalance in metabolism and immunological response.

The present study aimed to evaluate the radioprotective effect of bacterial supplements on the intestine and brain tissue of whole-body irradiated mice by analyzing the intestinal morphology, pyknotic neurons, and neuroinflammation.

## Methods

### Ethical statement

The ethical clearance for the current work was obtained from the institutional animal ethical committee (IAEC), Kasturba Medical College, MAHE, Manipal, India (IAEC/KMC/31/2019).

### Animal and experiment procedure

C57BL/6 mice (6- to 8-week-old, male) weighing 23–25 g were housed under controlled environmental conditions with temperature (20 ± 3 °C), humidity (50 ± 5%), light (12 and 12 h of the light/dark cycle), and continuous access to feed and sterile water. Animal care was carried out under standards established by the Indian National Science Academy in New Delhi, India, and the WHO in Geneva, Switzerland. After a week of acclimatization, animals were grouped into control (PBS), probiotics, radiation, and probiotics + radiation groups.

### Probiotics and irradiation treatment

Animals in the probiotics and combination group were orally dosed with known bacterial supplementation (Supplementary Table 1) containing 5 × 10^8^ CFU units in 200 µL of Phosphate Buffered Saline (PBS) daily from 0 to 14 days. Animals in radiation and combination groups were irradiated with a single whole body X-ray dose of 4 Gy on the 7^th^ day at a dose rate of 0.5 Gy/min (160 kV, 6.2 mA) using a small animal X-ray irradiator (Faxitron CP-160, CA, USA).

### Tissue collection and histology

The animals were deeply anesthetized and sacrificed by trans-cardiac perfusion with 0.9% saline. Intestinal and brain tissue were harvested and fixed in the fixative solution (10% neutral buffered formalin) for 48 h [17]. The cross-sectional plane of paraffin-embedded tissues was cut every 5 µm, and the sections were then transferred to slides coated with albumin or poly-L-lysine. These sections were further used for the analysis of intestinal morphology, intestinal integrity, neuronal survival, and neuroinflammation. For the intestine and the brain histological analysis, three animals per group were chosen, and for each animal, scoring was done by taking four to five sections. All the scoring was done using coded slides.

### Morphological analysis of GI tract: hematoxylin and eosin (H&E) staining

5 µm paraffin-embedded jejunum sections were used to perform the H&E staining. Briefly, slides were deparaffinized, dehydrated, and washed under distilled water. Further, the slides were stained with hematoxylin and counterstained with eosin. After subsequent washing and dehydration, slides were air-dried and mounted with DPX. A total of ten measurements per mouse were taken for the analysis [18]. Intestinal parameters like villi height, width, crypt depth, and mucosal thickness were measured using Image J software [19].

### Intestinal integrity analysis: periodic acid–Schiff’s (PAS) staining

PAS staining was performed using an adapted technique to examine the amount of mucus-producing goblet cells, representing the integrity of the GI tract [20]. Intestinal jejunum sections were deparaffinized, dehydrated, and oxidized with 0.5% periodic acid. After washing with distilled water, slides were incubated in Schiff’s reagent for 10 min in the dark (4 °C), and then the slides were placed in 0.52% sodium bisulfite solution. Further, the slides were washed in running tap water and counterstained with hematoxylin for 30 s. After subsequent washing and rehydration, slides were air-dried and mounted with DPX. For analysis from each treatment group, 30 well-aligned villi or crypts were scored.

### Neuronal survival analysis by Nissl staining

5 µm paraffin-embedded coronal brain sections were taken for the subsequent staining steps. After deparaffinization and dehydration, slides were placed in 0.5% cresyl violet solution for 1–2 min. After washing and rehydration, slides were air-dried and mounted with DPX. After staining, for the region-specific scoring, different brain regions were identified using the murine brain atlas [21]. The number of pyknotic cells in the CA1 (Cornu-Ammonis 1), CA2 (Cornu-Ammonis 2), and DG (dentate gyrus) regions of the three hippocampus sections were taken for scoring.

### Neuroinflammation by Iba-1 staining

For immunohistochemistry, 5 µm brain sections were collected on the poly-L-lysine-coated slides. After deparaffinization, dehydration, and washing, the slides were kept in antigen retrieval solution (citrate buffer, pH 6.0), further washed with wash buffer, and  kept in blocking solution. Further, the slides were incubated with primary antibody Iba-1 (1:500 dilution in 1% BSA) at 4 °C overnight. After subsequent washing, the slides were incubated with 3% hydrogen peroxide, washed, and incubated with a secondary antibody (1:1000 in 1% BSA). The color was developed by incubating the slides with 3, 3'-diaminobenzidine (DAB) chromogen and counterstained with hematoxylin. After subsequent washing and rehydration, slides were air-dried and mounted with DPX. Activated microglial cells in CA2, DG, and cortex regions of the brain were counted. Images of the histopathological sections were captured under 200× magnification using a light microscope (Olympus, IX-HOS, Tokyo, Japan). The images were analyzed using the Image J software [19].

## Statistical analysis

All the data were represented as mean ± SEM. A one-way ANOVA test with multiple comparison analysis was performed with the GraphPad Prism tool (v8.0, San Diego, California, USA). *P* < 0.05 was considered statistically significant.

## Results

### Radiation-induced intestinal morphology and integrity

**Fig. 1:**
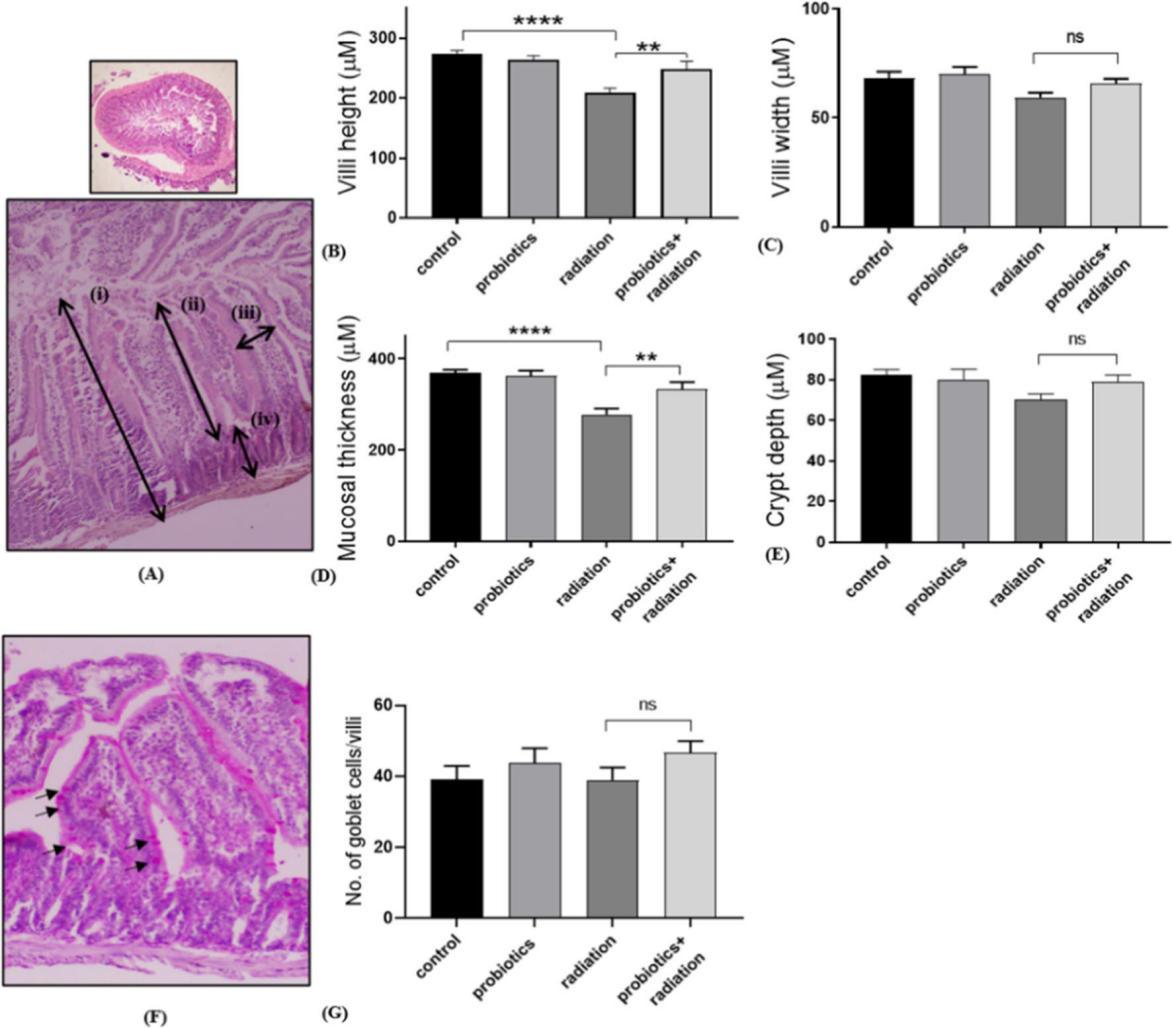
Analysis of probiotics effect on radiation-induced intestinal damage using H&E staining. (**A)** Representative images showing (i) mucosal thickness, (ii) villi height, (iii) villi width, and (iv) crypt depth. Graphs representing (**B**) villi height, (**C**) villi width, (**D**) mucosal thickness, and (**E**) crypt depth. (**F)** Representative image of PAS-stained villi (10×); the arrows show the goblet cells. (**G)** Graph indicating the number of goblet cells/villi. Data indicated as mean ± SEM (*n* = 3); *p* * < 0.05, ** < 0.01, *** < 0.001, **** < 0.0001, ns not significant

The intestinal morphology was intact in the control group, and there were changes observed in the other groups of radiation and the combination of radiation with probiotics. The probiotic treatment prevented the radiation-induced damage to villi height and mucosal thickness (*p* < 0.01). However, no significant effect was observed in villi width and crypt depth (Fig. 1). PAS staining did not indicate any changes in goblet cells or the integrity of the epithelial layer (Figs. 1F and G). Radiation exposure significantly reduced the morphology, while the morphology was retained in the combination group with probiotics.

### Radiation-induced neuronal death

**Fig. 2:**
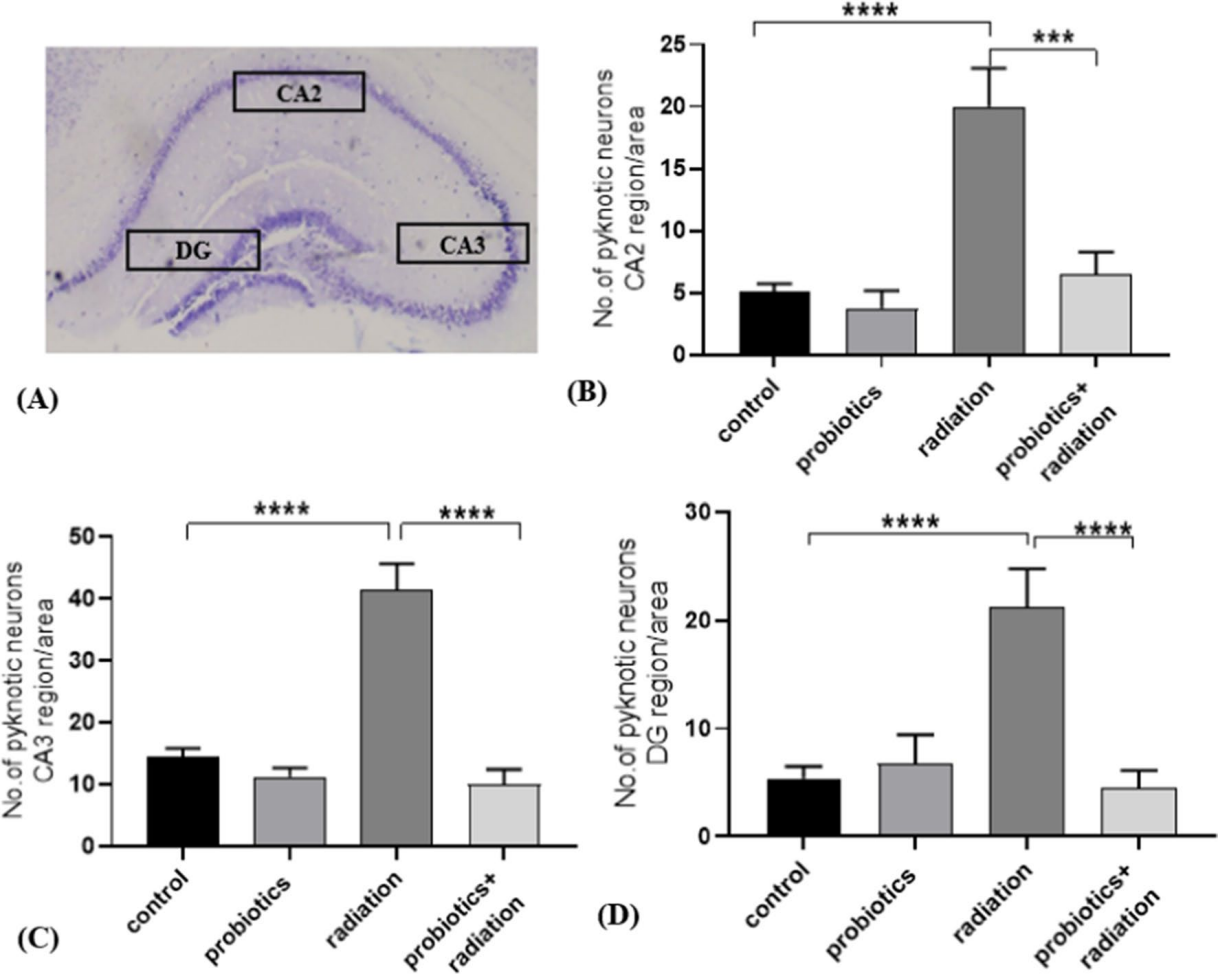
Radiation-induced pyknotic cells in the hippocampus and the effect of probiotic treatment. (**A)** The representative image shows Nissl-stained hippocampus. Number of pyknotic cells in the (**B**) CA2 region, (**C**) CA3 region, and (**D**) DG region. Data indicated as mean ± SEM (*n* = 3); *P* value ** < 0.01, *** < 0.001, **** < 0.0001

Radiation treatment could induce neuronal damage, through the induction of pyknotic cells and neuroinflammation. It is well known that the brain hippocampus is more sensitive to radiation exposure. Three different hippocampal regions were selected for neuronal survival analysis, including the DG, CA2, and CA3, responsible for memory and cognition. Radiation exposure significantly increased (*p* < 0.001) the number of pyknotic cells in the DG region. However, bacterial supplementation reduced neuronal cell death significantly (*p* < 0.0001). Similar protection was observed in both CA2 and CA3 regions in the radiation and combination group compared to the control (*p* < 0.0001) (Fig. 2). Neuronal survival analysis indicated that probiotics retained the number of surviving cells post-radiation.

### Radiation-induced neuronal inflammation

**Fig. 3:**
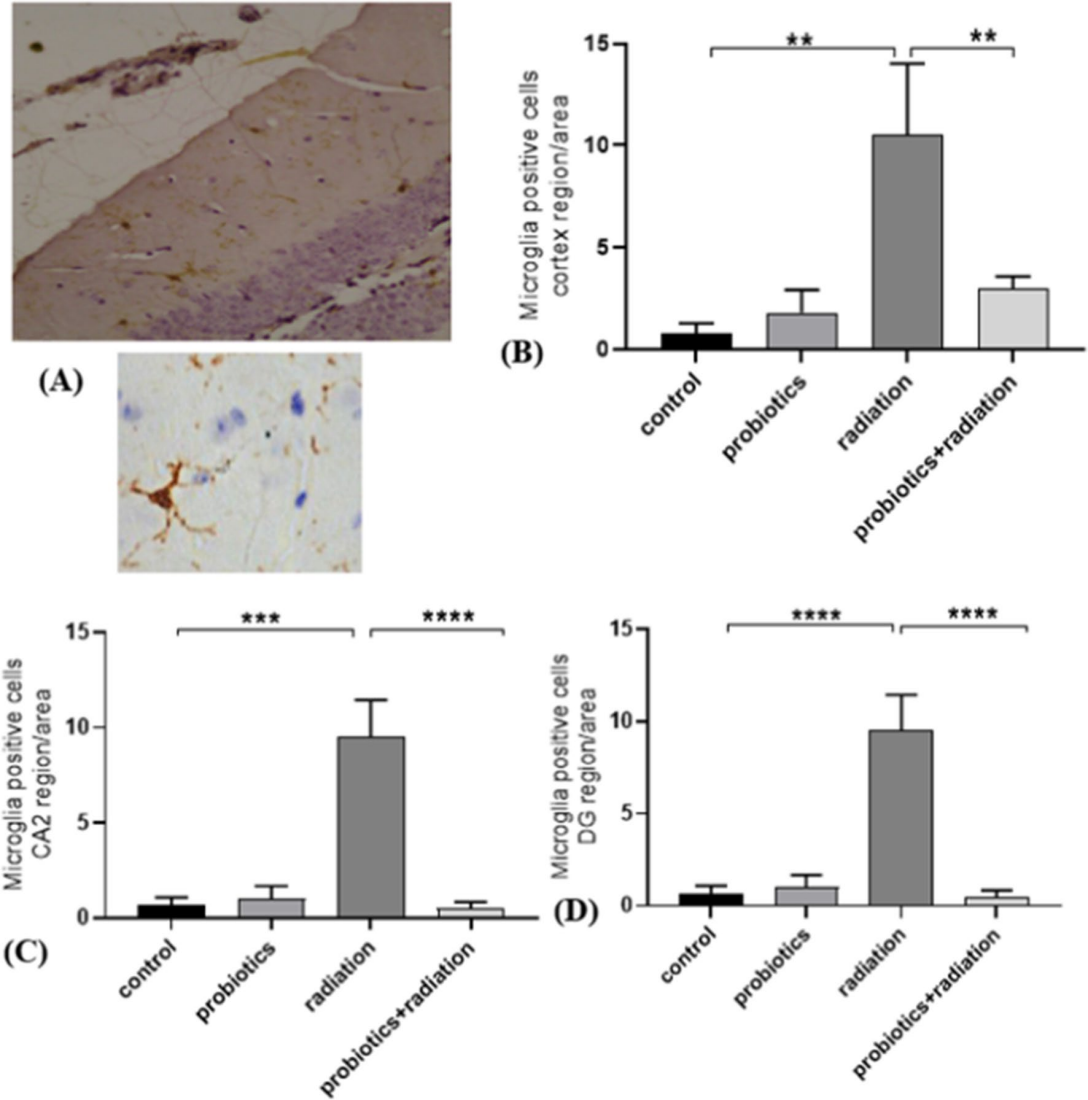
Radiation-induced neuroinflammation and mitigation by probiotics. (**A)** Representative image showing the microglia (Iba1-positive cells)-stained cortex. Graph showing positive microglia in the (**B**) cortex, (**C**) CA2, and (**D**) CA3 region. Data indicated as mean ± SEM (*n* = 3); *P* value ** < 0.01, *** < 0.001, **** < 0.0001

The number of microglial cells was scored in the cortex, CA2, and DG regions of the brain, which are mainly responsible for memory and cognition. In comparison to the control, a significant increase (*p* < 0.01) in microglial cells was seen in the cortex region of the radiation group, and probiotics prevented neuronal inflammation in the combination group (*p* < 0.01). Similarly, a significant reduction (*p* < 0.0001) of microglial cells was observed in the CA2 and DG regions of the combination group compared to the radiation group (Fig. 3). Altogether, the probiotic supplement reduced the neuroinflammation caused by radiation.

## Discussion

Unintended radiation exposure to normal tissue can cause cell death and affect tissue structure and function. Hence, there is a need to develop radioprotectors that are effective and non-toxic. The typical gastrointestinal injury occurs when clonogenic crypt epithelial stem cells die, resulting in the depletion of enterocytes, mucositis, secretory diarrhea, breakdown of the mucosal barrier, and dysfunction of various intestinal components including gut microbiota [22]. Gut dysbiosis further enhances the effect of radiation on intestinal tissue and also can impact distant organs like the brain [23]. In the current study, mice were administrated with oral probiotics and irradiated with a single dose of whole-body radiation, which successfully ameliorated the radiation-induced intestinal and neuronal damage.

The present study showed that the probiotic supplementation reduced radiation-induced villi and crypt damage and also retained intestinal integrity. In line with our study, multiple studies indicated intestinal tissue protection by probiotics containing  single species [24] or multiple species [25]. The observed protection may be by maintaining the regeneration and balance of the intestinal epithelium, as well as repairing intestinal damage and improving mucosal integrity. Probiotic bacteria could stimulate cytoprotective pathways in epithelial cells, resist ROS, shift pathogenic bacteria, and interact with tight junctions [24]. Additionally, probiotics may also have direct antioxidant effects, which can help protect against oxidative stress caused by radiation exposure [26]. Probiotics can also help repair DNA damage by activating DNA repair enzymes [27], stimulating cell growth, and promoting tissue repair and regeneration [28]. Further, certain probiotic strains have been shown to have anti-inflammatory properties and can suppress the radiation-induced immune response that leads to inflammation and reduce damage to the intestinal tissue [29]. Altogether, probiotics have a unique capacity to minimize the symptoms of GI problems that have been linked to neurodegenerative conditions. Further, evidences from preclinical research confirms beneficial effects of probiotics [30].

Ionizing radiation has been proven to change the structure of the mouse brain directly [31] or indirectly through the gut–brain axis [32], as well as induce neuroinflammation. Communication between microorganisms and the brain occurs through numerous channels, including the vagus nerve, certain neurotransmitters, SCFAs, cytokines, tryptophan compounds, and hormones [33]. The microbiota–gut–brain axis (MGBA) is a two-way communication system that links the host’s gut and brain functions [34]. Radiation can also increase neuroinflammation, with reports suggesting that radiation damages the neural precursor cells in the DG and disrupts hippocampal neurogenesis, which results in cognitive decline [35]. In the present study, we found that radiation has significantly reduced the neuronal cells in the hippocampus region of the brain, and the probiotics formulation showed the ability to reduce the number of pyknotic cells and reduce neuroinflammation by reducing the number of microglial cells. This observation is concurrent with the previous study where probiotics have been shown to modulate neuroinflammation [36] and facilitate damage reduction. Similar studies has shown that pre-treatment with a probiotic formulation reduced chronic stress-induced abnormal brain plasticity and improved neurogenesis [14] and also reduced oxidative stress in the brain by increasing antioxidant enzymes [34]. Further, probiotics have been successful in enhancing neurogenesis [37] and recovering memory deficits [38]. Recently, probiotic supplements were shown successfully to prevent and treat Alzheimer’s disease (AD) [39] and another study demonstrated the neuroprotective properties of the probiotic Lab4b in an AD-induced mouse model [40]. Significantly, *Lactobacillus *sp*.* supplementation has been shown to reduce neuroinflammation in mice [41, 42]. Rahmati et al. showed that probiotic supplementation in a cerebral hypoperfusion mouse model reduces neuronal damage in the hippocampus and improves spatial memory [43]. Interestingly, Xueqin et al. [44] showed that ProBiotic-4, a probiotic mixture, improves cognitive issues in elderly SAMP8 mice by modifying the microbiota–gut–brain axis, indicating that investigations specifically focusing on the gut microbiota may aid in the treatment of cognitive impairment. Based on the studies conducted in germ-free (GF) animals, now it is well known that bacterial colonization of the gut is essential for the growth and maturation of ENS and CNS and sensitivity to stress and anxiety-like behavior [34]. Also, recent studies in GF mice showed that the colonic epithelium’s neuronal innervation is diminished; nevertheless, microbial colonization can restore it. Moreover, enteric glial cells, which are crucial for maintaining neural networks and regulating gut homeostasis, are developed in mice by the regulation of their gut bacteria [34, 45, 46]. Though the exact mechanism behind the probiotics-induced recovery of neuronal damage is unknown, this process may be facilitated by the involvement of secretion of inflammatory molecules [36] from the supplemented microbial species, and hence, probiotics containing microbial species secreting beneficial metabolites [47] might prove to be a suitable formulation to combat neurodamage.

Thus, the existence of the gut-brain axis and the protective effects of the gut flora in both the gut and the brain opens up new possibilities for the development of novel therapeutic techniques for radiation-induced brain injury [48]. Interestingly, it has been noted that patients with brain tumors have a less diverse microbial environment [49]. Further, probiotics have been found to decrease cancer, particularly glioma, which accounts for 81% of all the malignant tumors of the central nervous system and is the most prevalent kind. Wang et al. demonstrated that *B. lactis* and *L. plantarum* could suppress the growth of gliomas in mice by changing the structure and metabolites of their gut microbiota and blocking the PI3K/AKT pathway [50].

The limitation of our study was that we did not look into the behavioral aspects. However, a radiation dose of 4 Gy in mice induced elevated anxiety [51] and impaired cognition [52]. It has been reported that radiation exposure results in abnormalities in cognitive function, unique learning, and memory impairments [53]. Further, the probiotics treatment showed reduced anxiety, depressive-like symptoms, and neuroinflammation [54]. However, behavioral alterations brought on by radiation are considered to be driven by the death of neural stem cells in the hippocampal region of the brain [51], which is one of the key observations of our study. Therefore, it can be surmised that neuronal cell death ultimately can give raise to behavioral impairments and, overall, probiotic supplementation can improve radiation-induced GI toxicity and reduce neuronal damage and neuroinflammation.

## Conclusion

The present study concludes that the given probiotics ameliorated the radiation-induced intestinal damage, neuronal damage, and neuroinflammation. Further studies looking into the mechanism of probiotics-induced recovery of neuronal damage and the involvement of the inflammatory molecules and microbial metabolites would be suitable for the formulation of probiotics to reduce radiation-induced neurotoxicity.

### Supplementary Information

Below is the link to the electronic supplementary material.

Supplementary file1 (DOCX 13 kb)

## Data Availability

The authors confirm that the data supporting the study findings are available within the article. Raw data are available from the corresponding author upon reasonable request.
