# Supplementary material for: Radioprotective potential of probiotics against gastrointestinal and neuronal toxicity: a preclinical study
Source: Clin Transl Oncol. 2023 Apr 18;25(11):3165–73. doi: 10.1007/s12094-023-03184-8 (PMC10514165; doi:10.1007/s12094-023-03184-8)
Supplement: Supplementary file 1 — Supplementary file1 (DOCX 13 kb) [file 12094_2023_3184_MOESM1_ESM.docx]

**Supplementary table 1:  Bacterial composition of the selected probiotics**

| **Bacterial species** | **No. of bacteria per dose** |
| --- | --- |
| *Lactobacillus acidophilus* | 200 million |
| *Lactobacillus rhamnosus* | 100 million |
| *Lactobacillus casei* | 100 million |
| *Lactobacillus bulgaricus* | 100 million |
| *Lactobacillus plantarum* | 100 million |
| *Bifidobacterium longum* | 100 million |
| *Bifidobacterium breve* | 100 million |
| *Bifidobacterium infantis* | 100 million |
| *Streptococcus thermophilus* | 100 million |
